# “Caring for a Crisis”: Care and Control in Community Mental Health

**DOI:** 10.3389/fpsyt.2021.798599

**Published:** 2022-01-13

**Authors:** Christien Muusse, Hans Kroon, Cornelis Lambert Mulder, Jeannette Pols

**Affiliations:** ^1^Trimbos Institute, Utrecht, Netherlands; ^2^Department Ethics, Law and Humanities, Amsterdam UMC, Amsterdam, Netherlands; ^3^Department of Social and Behavioral Sciences, Tranzo Scientific Center for Care and Welfare, Tilburg University, Tilburg, Netherlands; ^4^Department of Psychiatry, Erasmus Medical Center, Rotterdam, Netherlands; ^5^Antes, Parnassia Psychiatric Institute, The Hague, Netherlands; ^6^Department of Anthropology, University of Amsterdam, Amsterdam, Netherlands

**Keywords:** empirical ethics, community mental healthcare, psychiatric crisis, coercion and constraint, autonomy

## Abstract

In the debate on coercion in psychiatry, care and control are often juxtaposed. In this article we argue that this dichotomy is not useful to describe the more complex ways service users, care professionals and the specific care setting interrelate in a community mental health team (CMHT). Using the ethnographic approach of empirical ethics, we contrast the ways in which control and care go together in situations of a psychiatric crisis in two CMHT's: one in Trieste (Italy) and one in Utrecht (the Netherlands). The Dutch and Italian CMHT's are interesting to compare, because they differ with regard to the way community care is organized, the amount of coercive measures, the number of psychiatric beds, and the fact that Trieste applies an open door policy in all care settings. Contrasting the two teams can teach us how in situations of psychiatric crisis control and care interrelate in different *choreographies*. We use the term choreography as a metaphor to encapsulate the idea of a crisis situation as a set of coordinated actions from different actors in time and space. This provides two choreographies of handling a crisis in different ways. We argue that applying a strict boundary between care and control hinders the use of the relationship between caregiver and patient in care.

## Introduction

With the deinstitutionalizing of mental healthcare, there are concerns about how to care for a person experiencing a mental health crisis in the community (1, 2). In debates around this concern, care and control are often juxtaposed; care represents “the good,” whereas control is the evil to be avoided (3–5). In this article we take care and control as concepts that overlap in situations of psychiatric crisis. Care and control go together; or even care can be a form of control and control may be caring. We suggest the term *care-control* to analyze the relationships between the two. We use the metaphor of care-control *choreographies* (6, 7) to articulate differences. The metaphor of a choreography of a dance helps us to understand how care and control interrelate because it catches both the temporal and the spatial character of care practices around the onset of a psychiatric crisis.

To do this, we turn to the contrasting practices in two community mental health teams (CMHTs): one CMHT in Trieste (Italy) and one team in Utrecht (the Netherlands), and we explore how these practices relate care and control in different ways. This is interesting because the practices differ in the amount of coercive measures and the number of psychiatric beds. Some numbers: Trieste had 15 beds per 100,000 inhabitants (8) in 2018, vs. 89 per 100,000 inhabitants in the region of Utrecht (2017) (9). Each city uses a different accountability and juridical system. Trieste applies an open-door policy in all care settings and closed the psychiatric hospital (10), whereas in the Netherlands 41% of beds used for admission up to 1 year, and 19% of the beds on facilities for long stay are on closed wards (11). What can we learn from these differences? Which actors are involved in care-control situations in both sites? How does this lead to different care-control practices, and can we say something about the differences?

To answer these questions, we unravel the different ways in which crisis is understood and handled by adopting an empirical ethics approach in which the focus is on the practice of care and the values that come to matter in these practices (12–14). Ethnography is used as the main research method to examine these daily practices. We first sketch the two *care-control choreographies*, by showing how clients, professionals, and the specific care setting interrelate in the two teams. We then draw out the contrasts between the two choreographies. At the end of the paper we discuss if these alternative ways of understanding the relation between care and control can help in bridging the gap (3) between treatment on a voluntary basis on the one hand, and coercive measures on the other.

## Materials and Methods

### Ethnography as a Method

To answer the questions about daily care practices around a psychiatric crisis and the normativities embedded in these, we used an ethnographic approach with participant observation as the main method. Ethnography is chosen as a method because it offers the possibility of “studying at firsthand what people do and say in particular contexts,” (15) thereby allowing us to observe what is performed as the “good” (16) by those involved in care practices. Ethnography as a method is in line with the theoretical framework of empiric ethics that “analyzes ways in which people and things live together in particular practices as micro societies” [(13), p. 82] and the values enacted in these practices.

In this study, the first author conducted fieldwork in a CMHT in Utrecht for 5 months, divided in two periods. In Trieste she conducted more intense fieldwork in three blocks of for a total of 5 weeks. Although the first author has a basic understanding of Italian, in Trieste communication was aided by an interpreter who was familiar with mental health care, in order to get a detailed understanding of the daily practice. The first author (and interpreter in Trieste) joined workers on their daily routines, including home visits and team meetings. During the fieldwork the focus of the observations was not directed by preselected cases, but was informed by the research question about which ideas about good care are present in situations that were qualified as “the onset” of a crisis. In practice this led to a broad approach, in which not only patient-centered cases were studied, but also, for instance, the accountability structures in the teams.

During the observations, notes were made by hand, either on the spot (for instance during meetings) or immediately after (for instance, after house visits). More detailed fieldwork reports were written as soon as possible, usually the same day. Distinction was made between observational and more interpretative notes, which were an important part of the iterative character of the research in which analysis is not a separate phase following data collection, but part of the fieldwork.

Next to the participant observation as a method, interviews were held with three groups of respondents:

- (Care) partners of both CMHTs: selection of relevant care partners for an interview was based on the observational data collected. For instance, in Utrecht the fieldwork showed that there was frequent contact with the housing company and therefore they were approached for an interview [eight in Utrecht, four in Trieste, more interviews with partners were conducted in a previous study (10)].
- Clients of the teams: At each site clients were approached for a formal interview (three in Utrecht, four in Trieste) about their experiences with care and support from the CMHT. More importantly, with a larger number of service users there were frequent and differentiated informal forms of contact during the fieldwork; for instance, during house visits, meetings at the CMHT, lunch, or during visits to housing facilities or peer initiatives.
- Team members: next to the fieldwork some team members were approached for an additional interview (five in Utrecht, six in Trieste). The selection of these interviews was based on the iterative character of the research: specific observations led to additional questions and thus relevant team members were approached to reflect on these questions in an interview. An example in this paper is the interview of a psychiatrist in which the case of “Miss Westering” is discussed. Apart from these interviews, reflection on the daily care process with team workers was a continuous part of the participant observation; for instance, during travel from and to house visits.

At the end of the fieldwork, a group discussion with the team was organized at both field sites in which the initial results of the fieldwork were discussed and reflected upon with the team. During the fieldwork there was also an exchange between the two teams: the team in Trieste visited the Dutch CMHT and both teams, together with the first author, provided a workshop on the CCITP about crisis care (October 2018, Rotterdam). From the Dutch organization that the CMHT is part of there is a longer tradition of conducting visits to Trieste. Some of the workers from the CMHT in Utrecht, including the team leader, visited Trieste on at least one occasion.

### Position in the Field

Ethnography recognizes that researchers themselves are no neutral outsiders. The researcher is the one doing the interpreting, based on observations from a particular situated and embodied perspective. As Gibbons et al. (17) state, this makes reflexivity an important element of conducting qualitative research:

“*Reflexivity implies that the orientations of researchers will be shaped by their socio-historical locations, including the values and interests that these locations confer upon them. What this represents is a rejection of the idea that social research is, or can be, carried out in some autonomous realm that is insulated from the wider society and from the biography of the researcher, in such a way that its findings can be unaffected by social processes and personal characteristics*” [(17) p. 15].

To attend to the reflective character of qualitative research, it is important that the researcher is transparent about how the situated perspective of the researcher shaped the findings (18). In this study, the first author had experience with research in community mental healthcare, both in the Dutch setting and in Trieste. Results from previous research (10) informed the selection of research sites (Trieste and Utrecht) and the research question concerning dealing with crisis situations in a community setting. The fact that the first author was familiar with both research sites for a longer time made it possible to have easy and quick access to the field and aided the researcher in understanding what was going on. The first author is trained as an anthropologist and therefore could observe the daily practice of care and decisions made with relative distance, while, still being familiar with the organization of care and most of the language used in the teams, as well as the more specialist medical descriptions.

### Analysis of the Material

As stated above, in ethnography the analysis of data is not a distinct stage of the research (17) but a continuous process in which the researcher goes back and forth between empirical and theoretical informed questions and the data collected. After the fieldwork was conducted, both interview transcriptions and fieldnotes were analyzed using Maxqda (2020). The first round of analysis was open: the material was read and discussed by the research team and reread by the first author and a first selection of important themes was made, such as ways of preventing a crisis. The next stages of analysis consisted of a combination of open and selective stages to sharpen the analysis (constant comparative method). This led to a focus on the relation between care and control. In the analysis, we attended to both the similarities and differences between Trieste and Utrecht.

During the analysis we chose to use the metaphor of a choreography (6, 7) to describe the way different actors interrelate in moments of a so-called psychiatric crisis and how different forms of care and control are part of this. Law uses this metaphor to describe the complexities around caring and killing in the context of the foot-and-mouth epidemic among cattle in 2001 in the UK. Law (7) refers to Cussins (6) when he describes a choreography as “the arrangement and distribution of events and actors in space and time, sometimes bringing actors together and sometimes keeping them apart” [(7), p. 67]. Law points out that in the literal sense the term choreography refers to the writing of a dance, but that in common practice “the term is used to refer to a space-time set of rules or practices which shape but do not determine the actions of the bodies and dancers”[(7), p. 68]. We use the term *care-control choreography* as a metaphor to encapsulate the idea of a crisis situation as a set of coordinated actions between different actors in time and space. By contrasting the *care-control choreographies* of Trieste and Utrecht we will see that many of the “actors” entering the scene in both CMHTs are comparable; however, what is enacted, when, by whom and where differs.

### Ethics

During site visits and meetings, the first author was always open about her role, and in the waiting area and hall of the CMHTs information about the research was provided, including a picture of the first author and her contact details. Respondents for interviews gave their informed consent. All material was anonymized, and no names or other personal details were collected. Following the anthropological tradition, pseudonyms are used in this text and some personal characteristics are changed when this was necessary to protect the anonymity of the persons involved. The METC from VU University (FWA00017598) has declared that the Medical Research Involving Human Subjects Act (WMO) does not apply to the study. Additional ethical permission was provided by the ethical commission of the Trimbos-institute.

Different strategies were used as a member check. First there was the group discussion in both teams. Additionally, if agreed upon, interview transcriptions were sent to the respondents. Respondents were also informed about quotes used in this article, whether it be fieldwork descriptions or part of an interview. Some key contacts in the field were offered the chance to read the whole article before submission and offered their comments and insights. This did not lead to substantial changes.

## Results: Two Care-Control Choreographies

### Background

#### Historical Background

The “Trieste model” of mental healthcare that has developed since the 1970s is based on the ideas of Franco Basaglia (1924–1980), an Italian psychiatrist. He stated that the person with the mental illness—and not the disorder—should be placed at the center of the mental health system. In the 1970s he proposed a different way of organizing Trieste's mental health system: closing the psychiatric hospital and making a radical shift toward organizing mental health care in the community by starting Community Mental Health Centers (CMHC). Important principles in this movement were offering a low threshold to care, working with open doors and minimizing coercion (19, 20). This movement in 1978 led to the implementation of Law 180 in the whole of Italy, which called for the closure of psychiatric hospitals. The actual implementation of this law varied greatly between the various regions of Italy (21, 22).

In the Netherlands, the process of deinstitutionalization was more gradual. Different forms of community mental health were already in existence before World War II and served as an example for other countries at that time (23). In the different phases the deinstitutionalization process in the Netherlands went through, the aim was to reduce the number of beds in psychiatric hospitals and enlarge social inclusion, rather than closing the hospital entirely. Psychiatric hospitals now function in cooperation with CMHTs and Flexible Assertive Community Treatment teams (24, 25) and other forms of ambulatory care.

#### CMHT Trieste

Trieste is a city with 205,000 inhabitants in the north of Italy. Each CMHT consists of nurses, psychiatrists, psychologists, rehabilitation specialists and social workers and is located in a Community Mental Health Center (CMHC). The CMHC functions as a single point of responsibility in a catchment area, provides day, office-based and home treatment, and is a drop-in center for service users, neighbors, family and others. Nurses take turns to staff the reception, enabling them to act quickly on demands for care both from patients themselves or others. There is no waiting list and there is no need for a referral to receive care at the CMHT. In the center where the first author conducted observations there was a total of six beds in one-person or two-person rooms for people who needed to stay overnight. If people are in need of acute psychiatric care after 8:00 p.m., they are referred to the psychiatric crisis department at the general hospital (SPDC- Servizio Psichiatrico di Diagnosi e Cura -psychiatric service for diagnosis and treatment), which has a small acute ward with six beds. Both the CMHC and the psychiatric ward have an open door policy.

The CMHT works together in projects with different social cooperations, which provide supported living and sheltered housing, and with other care providers like social services that operate in the same health district. The CMHT has the aim of responding to a crisis in the community, and tries to avoid transitions in care by providing care in the community and by avoiding acute hospitalization (26, 27).

#### CMHT Utrecht

Utrecht is located in the middle of the Netherlands and is a somewhat larger city than Trieste with approximately 360,000 inhabitants. The CMHT where we conducted our fieldwork consists of care workers from two organizations; one aimed at supported living and the other providing mental health care. A proportion of the patients in the caseload of the team receives care from both organizations. Staff include a psychologist, a psychiatrist, an expert by experience, mental health nurses, and personal case managers. In their work, the CMHT adapts the model of Flexible Asserive Community Treatment (FACT), a care model that combines individual case management with shared caseload and assertive outreach. In contrast to Trieste, where a referral is not required for care from a CMHT, the team provides care for those that are indicated as being in need of *specialized* mental health care treatment. If there is no indication for treatment or problems are not primarily psychiatric, people are referred to other teams or care domains. The mental health care landscape in Utrecht is thus both more differentiated and fragmented than in Trieste: next to the CMHT there are teams for first-line treatment, teams organized around a specific diagnosis (e.g., Autism Spectrum Disorders) and there are different clinical facilities. Some of them are run by the same mental health organization, while others are located in the general hospitals in the city.

### Care-Control Choreography in Trieste

What situations are seen as a risk for (the onset of) a crisis both in the CMHT of Trieste and in Utrecht? We start with the care-control choreography in Trieste. We take the care around specific service users and situations as a starting point to show how service users, professionals, and the specific care setting relate to each other.

#### Identifying a Crisis

How is a crisis defined and identified in Trieste? This is a recurrent theme at the team's daily meetings. A head of a CMHC describes a crisis as follows:

*Team leader: A crisis is often not the crisis of a person, but the crisis of a context. If there are good relations in the network or family, it's easy to solve problems. Often the relations are not good and then the problem goes in circles, it maintains itself*.

*Interviewer: What about psychiatric symptoms?*

*Team leader: Those problems are there and they are real. You shouldn't deny that, but it's not so much about symptoms themselves, but about symptoms creating difficult behavior. Symptoms are always in a relation where the problems evolve: in the system (Interview, head of CMHC)*.

If a crisis is seen as a crisis of a context than different actors enter the stage: next to mental healthcare, there is the family and the broader social network. They are needed to identify the onset of a crisis:

*If we talk about the set-up of a crisis, and to intervene at the right moment, it is crucial to be able to listen to the people. Everybody can hear screaming or crying, that is not so difficult. But if someone is whispering you should be able to hear it as well (Interview former-director Trieste CMHC)*.

Crisis may start with a whisper that may be hard to hear for team members. To hear these whispers the team needs a strong connection with the social network of service users. Identifying a crisis is hence a shared endeavor of the CMHT and the broader social network. The team finds it important to discover the signs of a crisis early on, and to achieve this, the social network is involved as much as possible (28).

#### Caring and Controlling for Riccardo

Here is the situation of Riccardo, a young man who stays at the center during the first period of my fieldwork:

*When I enter the CMHT's garden together with Arianna, a nurse, Riccardo sits there, smoking, another nurse next to him. Arianna explains that team members always join him when he goes outside because of the risk of him wandering off. She tells me a bit more about his situation. Riccardo came to stay at the center on a voluntary basis a few days ago because there was a “crisi brutta” in which he became physically aggressive as well. He is a young man in his early twenties, but has already been in the care of the CMHT for a couple of years. She states that one of the problems is his relationship with his parents; they were never supportive of treatment or medication. They tried different things—to start an education, to find a job—but it never worked out*.

*During an evening shift a male nurse describes the attitude of the team towards Riccardo as finding an equilibrium between keeping an eye on him and not being too close. I observe an example the next day: a volunteer of a youth organization that they involved in the support of Riccardo takes him out for an ice-cream, in a trattoria down the road. That same afternoon a nurse walks with Riccardo towards the gate of the garden, announcing “We're going for an ice cream!” “But we did that already today!” another nurse replies. “O.K., a coffee then!' And off they go (based on fieldnotes)*.

In this situation there are different actors in the care and control of his situation. First there is the center. Because the CMHT is in a location with six beds, there is the possibility of admitting Riccardo to the center without transferring the care for him to a separate clinical team. In line with the philosophy of Basaglia, in the center the doors are always open. Yet this does not mean that the movements of Riccardo are not controlled in some way. Instead of a door keeping Riccardo inside, the nurses and others (volunteers, or even the first author by answering the often repeated question “Where is Riccardo?”) are involved in keeping an eye on Riccardo and prevent him from wandering off. The staff sits next to him smoking in the garden, and take him outside for an ice cream or a coffee. This caring for Riccardo is at the same time a way of checking and controlling his movements, guiding and going with him to places where he wants to be, rather than forcing the wishes of the team on his movements. Driessen has coined this way of aligning the wishes of patients with the wishes of professionals as “will-work” (29).

A closed door controls the movements of patients, but caring and staying close can be understood as forms of controlling movements as well. But they are not the same. A closed door restricts movements by force, and separates those from inside from those outside. Guiding and following movements does something else; it controls movements by engaging in intense contact and staying close. Although this can be directive, the course of the activities is not as determined as if Riccardo would have been behind a closed door. Different negotiations and ways of “being looked after” are possible.

#### Crisis Care at the SPDC

Guiding and following movements without a closed door works on the psychiatric ward of Trieste's general hospital as well:

*I join the psychiatrist who is on duty on the late afternoon/evening shift in the SPDC. An ambulance has brought in a young man from the refugee shelter located in Trieste's harbor. He was intimidating people, acting violent and self-harming. When the psychiatrist wants to examine him, the man first does not want to leave his room. Sometime later the man is walking through the corridor in the direction of the exit. He has a bandage around both arms. The psychiatrist and two nurses follow him, one of them blocks the direct access to the door by taking a shortcut through the administrative office. The psychiatrist continuously tries to engage in a conversation with him in a mix of Italian/English during their tour through the hallway, persuading him to stay for the night: “Where would you like to go at this moment of the day? You are sick, please stay for the night.” “Really you are too weak now, come on, you have to rest a little” and “tomorrow you can leave, but please rest now- per favore, per favore.” The psychiatrist leads him back to the living room by giving him an arm. This process is repeated twice. Formally, he has been admitted voluntarily, so he has the right to leave the ward. The psychiatrist confirms this, but keeps persuading him to stay. She tells him, “Of course the door is open, if you want you can leave. But really, it is wiser if you stay for the night. You want to smoke? You can smoke in your room!” Then the man returns to his room and the ritual repeats itself again. The psychiatrist offers him medication with the explanation that “this will make you calm,” which the man accepts. Still, he wants to leave, stating that he has an appointment. The nurse offers him the use of their telephone in the administration office to arrange his appointment. In this little office the psychiatrist and the man sit down, and she tries to engage him in a conversation again: “You are so young. What age are you? Twenty? Please sit down, you are in no condition to go,” and she points to the bandages around his arms. Again she leads him to his room, linking arms with him. They walk down the corridor together; it appears the man is staying for the night (based on fieldnotes)*.

In this situation, the young man is persuaded to stay for the night because the care professionals found the condition of the man too severe to be out on the streets. They try to control the situation by persuading him to stay, by positioning themselves and by moving into the space to make his exit more difficult. The most important instrument to achieve this is to engage him in a conversation, and in doing so, looking for opening points that they can use in their negotiation with him. He is allowed to smoke in his room for instance, though officially this breaks the house rules. They let him use the telephone and at the same time grasp this opportunity to sit down with him and to have a conversation. They argue, plead, cajole, and almost beg, but never directly force the man to stay. The physical characteristics of this ward—the open door—creates a situation in which the only way to make him stay is to engage in intense contact.

Next to the efforts to engage in a conversation and intense contact to control the situation, the man is made to stay by moving through space in specific ways, without confronting him physically in a direct way. Indeed it looked like the performance of a dance, where each partner moves in relationship to the other. The psychiatrist physically performed this move by giving him an arm and leading him to the desired location: his room. Once again controlling movements are performed by guiding; gestures, moves, and ways of touching each other.

#### Medication as Care-Control

Another part of the care-control choreography in Trieste is offering medication. Offering medication is part of the negotiation between professionals, service users and sometimes the family, as is the case with Riccardo. Medication is a form of care that sometimes needs to be controlled, even if not forced (i.e., checking whether medication has indeed been taken). Yet this controlling is in itself a way of preventing escalations. Many service users come by the center to pick up their medication daily, monthly, or anywhere in between. To have people come over for medication on a regular basis is a combination of caring (by medication) and controlling by checking how the person is doing. It offers the team the possibility to intervene immediately when something seems wrong:

*Nurse Mauro is on his way to Ravi, a man who lives with his mother. Ravi usually visits the center every morning to pick up his medication, but made a call that he wasn't able to come due to a backache. For Nurse Mauro this is a reason to do an unscheduled check-up visit. When we enter the apartment, the mother leads us to the kitchen; Ravi is there, sitting on a wooden bench. Mauro asks how he is doing. Ravi complains about his back and his fear of not being able to move anymore. The mother constantly enters the conversation, explaining how heavy the situation is for her. Mauro asks the mother about her family. The mother welcomes the chance to show photos of the family and the woodwork of her deceased husband. It all takes more than an hour. During this conversation Mauro hands over the medication to Ravi: pills and a fluid, one with P (“pomeriggo”/afternoon), one with an S (“serra”/ evening). On the way back I check if it is extra medication. “No,” says Mauro, “but I took it since Ravi didn't visit the center this morning.” He states that this was a good morning and I ask why. “because there was time to talk,” he replies. “This talking is not acute at the moment”, Mauro adds, “but it is of importance in the long term, to prevent a crisis” (based on fieldnotes)*.

Distributing medication in this way can be understood as part of the care-control choreography since it offers the opportunity to check how service users are doing, keep their medication intake stable, and build relationships with the family in order to intervene quickly when necessary.

But the check on medication works in other ways as well. In an interview the director of the MH services points out that medication is part of the relationship between service users and professionals. “*Sometimes you have to accept that people refuse medication. The acceptance of medication is often an important step in the larger process towards working on recovery.”*

Lastly medication can be a way to enable a relationship or conversation. This happened in the SPDC; offering the man calming medication made it easier to engage him in a conversation despite his agitated state. As one of the psychiatrists stated in a conversation about controlling a crisis, “S*ometimes it is first sleep, then talk!”* Medication, than, opens up ways to enable a relational approach to care. Medication thus is part of the dance around dealing with a crisis and not an isolated intervention.

#### The Role of the Network

Time to talk—whether this is about woodwork or medication and symptoms—is important in the long run because the aim of the Trieste choreography of caring and controlling is to build a relationship with both the patients and their social networks, such as the mother of Ravi. This relational embeddedness is important to prevent a crisis. Working on relationships and creating a network could also be witnessed during Riccardo's admission in the center. The staff established contact with the volunteers of a youth organization in the hope that this would create new contacts, involved a social cooperation in their work and tried to find housing together with other young people. Crisis work in these situations works on relationships by building and maintaining the network. An former director of the MH Trieste reflects that:

*The concept of a crisis in itself is non-existent, it is always in a specific context. And as a professional it matters what you do in that context. There is always a set-up and if you are organized in the local community then you can intervene in every step. Often, when we call something a crisis, we only see the end of the process, the acute moment. But if you are truly present in the local community you can intervene before that phase and you can make a difference (Interview former- director MH Trieste)*.

The realization that a strong social network can not only prevent but also buffer and thus control a crisis means that a lot of the work in Trieste is dedicated to building and maintaining these relationships (30). The network can a be a source of information during a crisis. Contact with the social network creates a care-control network of “many eyes” in which it is easier to check how one is doing, to “hear the whispers” in the build-up to a crisis and to intervene if necessary.

#### The Juridical System

In the situations with Ravi and Riccardo, although contact was sometimes difficult and required a lot of work, the treatment was voluntary in the sense that the situations were controlled without legal measures and without the use of direct force or coercion. To avoid coercion, professionals engage in negotiations, persuading patients to accept care. If persuading, negotiating and involving the network does not work and the situation is perceived as severe, a community treatment order (CTO, TSO in Italian) may be issued, based on the need for treatment criterium. The absence of a dangerousness criterion relates to the vision of Basaglia, and it is seen as a fundamental step to break the often-made connection between mental disorders and dangerousness (31). In Italy the dangerousness criterion is not listed as a requirement for forced treatment (32). The need for treatment criterion prevails. The law stipulates that within a TSO doctors are obliged to seek consent and in that case the involuntary treatments ends.

In Trieste the number of TSOs issued, however, is relatively low, in 2018: there were 30 TSO's for 18 people (8). If a TSO is issued this is done mostly in a center to avoid transitions in care as much as possible. This means that nurses and others are assigned to support and guide a person with a TSO (even side-by-side when the crisis is severe) in the center and to join them going outside. When a TSO is issued, often different actors are involved to make this intense support possible. These may be relatives, people working for social cooperation's or others within a patient's network.

### Care-Control Choreography in Utrecht

#### Identifying a Crisis

To understand how in Utrecht the choreography of care-control takes shape and how it contrasts with the care-control choreography in Trieste, we must examine how situations at risk of a crisis are identified. Therefore, it is important to describe a specific instrument that is used in the CMHT in Utrecht: the FACT board.

The FACT board is an excel sheet that is projected on a screen every morning in the team meeting. The excel sheet lists clients who are perceived as being at risk of a crisis. The “board” sheet provides information about the diagnosis, the reason someone is “placed” on the board, along with details about their social network, drug use, juridical status, and the goals and wishes that were formulated together with this client. Every morning possible interventions are discussed, such as adjustments in medication, applying for a juridical measure or intensifying the frequency of house visits. The idea behind the board is that it offers a flexible way to shift between daily team work for those (at risk of) being in crisis, and a less intense, individual case management approach in periods when someone is more stable (24).

In the CMHT Utrecht, “being placed on the FACT board” thus means that someone is identified as in crisis or at risk of a crisis, based on the contact with the person self or with the network. This can be down to a number of different reasons. On a random morning the first author listed the reasons why service users were placed on the FACT board on that particular day. This shows a great diversity of social and medical reasons:

*Raising of agitation and suspicion, self-mutilation/Expression of suicidal thoughts/Low body weight/Aggression, engaging in drinking/Anxiety, (2x)/Superstitious, intimidating behavior/Just discharged from an hospital admission/At risk of the child being taken away/Weird, compulsive behavior/At risk of eviction (fieldnotes)*.

The board offers a structured way of identifying the risk for a crisis when it is more or less acute. Once a situation is identified as at risk of deteriorating into a crisis, how is the situation controlled and cared for? Here is the case of Miss Westering, a woman in her 40s, who lives together with her husband and two children.

*I first hear about Miss Westering during an extra meeting that was scheduled because the team is worried about her condition. Without consulting the psychiatrist, she stopped taking medication and the team is afraid she will be hypomanic. Her husband says she is hallucinating. They discuss how they can break the repeating cycle of quitting medication and ending up in a crisis again*.

*The next week a nurse updates the team that Miss Westering called the crisis team and an ambulance twice at night. The team knows from experience that she will stabilize if she starts taking medication, but so far she has refused. What to do? Start supervised medication intake or start a juridical procedure to force her to take medication? A nurse explains to me that providing supervised medication intake is done by another service provider that also works outside of office hours. Another nurse states that they have to be strict and clear because there are children involved. We have to say “This is what we are going to do!”*

*When the meeting has ended, it turns out that Miss Westering's husband is waiting in the CMHT office. He came to the CMHT to ask for help because he didn't sleep the whole night; he was watching over his wife, afraid that she would wander off. They decide to pay her a home visit. When the team returns they tell me that the situation was severe, and that they want to hospitalize Miss Westering immediately with an emergency involuntary admission (EIA). The next day a case manager tells me that when they came to her house she had already packed her bags; Miss Westering was willing to go to the hospital. She is now at a crisis ward on a voluntary basis (field notes)*.

In the case of the care-control for Miss Westering, different actors played a role. First there is the CMHT. When a situation around a patient in their care is identified as a crisis, both care and control around a service user is intensified. Just as in Trieste, more team members are involved in a flexible way, and every team member is updated about the situation through the FACT board. Since the team in Utrecht consists of both workers from a treatment organization as well as an organization providing supported living, this also offers the possibility to intensify care by involving the latter. In contrast to Trieste, a hospital admission in Utrecht may be seen as a good intervention to control the situation and care for the client. More intense treatment and support can be given than the CMHT can provide on an ambulatory basis, for instance when someone is seen to be in need of 24/7 care, which the CMHT in Utrecht does not offer.

Hence, different care partners and different forms of expertise are involved in the care control choreography for Miss Westering: there is a network of different types of professionals and care organizations that enter the stage when a crisis is suspected and the CMHT perceive the situation as risky. A separate organization may be called upon when supervised medication intake seems necessary. In addition there are the emergency services, and as a last resort there is the crisis ward, where clients can be admitted either voluntarily, or against their will with a legal measure. Different from Trieste, continuity of care from the CMHT in Utrecht does not always mean providing care by the same team (28), but connecting responsible organizations functioning in a network to provide continuity of care. Rather than staying in the care of the same team, in Utrecht a crisis admission means a transfer to a clinical team, and care is coordinated between the two teams and forms of expertise.

#### The Role of the Network

Next to the CMHT and other mental health facilities the social network of clients such as Miss Westering is also an important factor in the situation. In Miss Westering's case her husband supports her but also controls her safety by staying up all night to watch over her. Then there are the children. Their vulnerability is a reason for the team to pay extra close attention and in this way they influence the care-control for Miss Westering. This becomes clear during a morning meeting during which the psychiatrist shares her experiences:

*The psychiatrist talks about a home visit to Miss Westering earlier that week. During the house visit the psychiatrist mentioned that they might apply for a community treatment order[CTO- supervised treatment], but Miss Westering did not show any reaction. The psychiatrist then talked about the children, that it was important for her to be a strong mother. She shares with the team that she hesitated whether this was the right thing to do and that it felt a bit manipulative. A nurse says, “Now you are being too hard on yourself, it is the truth, isn't it? Negotiating is part of our work” (fieldnotes)*.

We reflect on this in an interview. The psychiatrist explains more about her considerations:

“*I found it difficult. I prefer to discuss openly and rationally with someone about what is going on and what would be a wise choice and to leave as much autonomy to the patient as possible. But on the other hand, it is part of our daily work to cajole people a bit in the direction of those choices we find healthy or wise. It has two sides; I like to be open and direct, and this {to refer to being a good mother CM} felt a bit like manipulation” (Interview, psychiatrist)*.

The children become part of the care-control choreography when the psychiatrist involves them in the discussion with the woman about taking medication. This is a dilemma for her: when does persuasion become manipulation? Ideally, she respects the autonomy of patients and she openly discusses the different treatment possibilities on the principles of shared decision making. But when such a conversation is not possible, negotiation, or persuasion to avoid further escalation is also part of the job. The problem here is that this care vision based on individual autonomy makes her wonder if engaging in persuading or manipulating is still good care, while acknowledging that it is part of the daily care practice. In Trieste, negotiation and persuasion were not problematized in this way, but rather they were seen as a legitimate way of avoiding coercion from within the relation.

#### Medication as Care-Control

In the care-control for Miss Westering, medication plays a role in different ways. First, the lack of motivation to continue taking medication is seen as one of the reasons to identify the situation as “at risk.” It is not only identified as a risk because medication adherence is seen as important to prevent a crisis in general, but specifically because they know from the history of Miss Westering that quitting medication increases her risk of a crisis. The ideal of the psychiatrist to openly discuss different possibilities about the use of medication and side effects and together come to the best solution does not seem to work. This means that other ways of care-control are employed. If negotiating and persuading do not work, another possibility comes to the fore: forced care.

#### The Juridical System

The fieldwork was conducted 1 year before a new law concerning forced care was implemented in The Netherlands in 2020 (33). In the case of miss Westering, the old law was still applicable. In Miss Westering's case this meant that two forms of forced care are discussed. First there is the community treatment order (CTO/rechterlijke machtiging in Dutch) that is mentioned by the psychiatrist on her home visit to Miss Westering. A CTO is a juridical status at the time of the research that can be applied in a non-acute situation. The CTO contains directions for the client to stick to certain conditions, such as keeping in contact with a psychiatrist or adherence to a course of medication, to avoid forced hospitalization. This CTO thus makes it possible in an ambulatory setting to use a certain force to make sure that service users acquiesce to these rules without direct coercion being applied. It is seen as “*stok achter de deur”* (literally, a stick behind the door), a kind of safety net that can be used in case someone does not stick to agreements made. The “CTO” was frequently mentioned in the team as an instrument to align the behavior of clients with the wishes of the team. It was perceived as a way to avoid coercion, while in fact it is part of the law concerning forced care. This dual character of the CTO was discussed in an interview with a nurse:

*Nurse: We often refer to it as a stick—a “stok achter de deur.” It is not really coercion—I mean, it's not like—you do not take those pills, therefore… Interviewer: But it is a juridical measure… Nurse: Yes, of course, but in my opinion, even if one doesn't stick to all the conditions you still have to engage in a dialogue. It is not like you do not stick to one of the conditions so immediately you are admitted to the hospital. Interviewer: It does not work like that… Nurse: No, only... It is really about one's safety or the safety of others, rather than “you have to” (interview mental health nurse)*.

The nurse stresses the relational character of working with this measure; it allows the team to engage in a dialogue with the client in a way that stresses the urgency of the situation. It relates to the dilemma often raised in teams of whether one can intervene when someone refuses care. Again, proceeding from the paradigm that the patient is an autonomous individual and has the legal right to self-determination, care providers ideally are open and transparent and discuss the different treatment possibilities (34). But this becomes problematic when people refuse care or even refuse to engage in such a dialogue. From the ideal of individual autonomy the option to intervene without having met the criteria for forced care is seen as problematic (35). Here the dilemma is solved by a juridical back-up for intervening when a relational approach fails.

When the situation of Miss Westering worsened and her husband came to the center in desperation, the emergency involuntary admission procedure was mentioned (EIA/IBS in Dutch). This EIA procedure is a short-term measure for acute and immediate admission and care. It is a way to admit someone to a psychiatric hospital in case of acute danger. This was seen as necessary when they visited Miss Westering that morning; but before it could be issued it was abandoned, because Miss Westering decided to cooperate with a hospital admission.

Miss Westering's situation shows how juridical measures are not only a way to apply forced care but also function as instruments in the relationship with the client to persuade and negotiate. As the use of the conditional CTO shows, the distinction between juridical forms and relational forms of control in practice are not always clear-cut.

### Contrasting the Two Choreographies

Above we described the care-control choreography around a crisis for both Trieste and Utrecht. Which contrasts are there to be made?

#### The Start: Identifying a Crisis

In Trieste, a crisis is defined primarily as a crisis of the social network. This has consequences for the way the care-control choreography is shaped; building relationships and strengthening the social network of service users is an essential element in the care-control choreography around a crisis. By building relationships the health services, social cooperation's, family, and others are all connected, and these connections can help not only to care for a crisis, but to control it as well. This is why working on relationships and engaging in a dialogue is seen as essential.

In Utrecht, a relational approach is applied as well, but situations are primarily defined as a crisis of the individual, may it be due to medical reasons (e.g., an intensification of symptoms), or more social reasons like being at risk of eviction. Although the network can play an important role in a crisis situation (as we saw in the case of Miss Westering), the care of the team is directed to the individual.

#### The Dancefloor: One Center or Different Places and Expertise

The CMHT in Trieste is located in a center which offers different possibilities and restrictions to care-control a crisis than the CMHT in Utrecht. The CMHT in Trieste has the possibility to (voluntarily) hospitalize service users with a low threshold in the center without waiting lists, and thus has the ability to offer care 24/7, avoiding discontinuity of care by transferring someone to a clinical facility. People can also visit the center as a day hospital, come there to pick up medication or eat lunch. All these possibilities give the team the opportunity to care-control by observing and reacting quickly if something might seem amiss—like Ravi having a backache. On the other hand, the team has limited possibilities to refer patients to more specialized forms of care; there is no crisis team and only a small psychiatric ward of six beds.

In Utrecht the CMHT is not a direct access point into the care system for people in need of care. The CMHT does not operate from a center, operates during office hours, and is embedded in a differentiated care landscape consisting of different specialized teams to which people can be referred (24/7 crisis team and different options for voluntary and involuntary hospitalization). The CMHT in Utrecht thus needs a strong cooperation with other professional care partners. Continuity is created not by continuity of caregivers as in Trieste, but by connecting different teams and expertise in a successful way.

#### The Dance: Restricting and Guiding Movements

Both choreographies show that in controlling and caring for a person in a crisis, restricting movements can be important. But the way this is done in Trieste and Utrecht differs considerably. The open-door policy of Trieste has shaped creative ways of moving along with clients: accompanying and guiding movements, staying close, and moving in and outside the center in a non-coercive way (e.g., going for ice creams). In Utrecht, following a person's movement is not part of the daily practice of the CMHT. Restriction of movement takes the shape of hospitalization as a way to control the situation and care for the client. At that point a patient is admitted (voluntarily or not) on a (closed) ward. The transition between freedom of movement and restriction by closed doors thus is more radical in Utrecht compared to the relational way of aligning movements in Trieste, in which a strict form of coercion is avoided and ways of guiding movements can be more or less intensive.

Controlling movements can also be done by applying for juridical measures; in both Italy as well as the Netherlands this step only becomes possible when all other possibilities of voluntary care have failed. There are two important differences in the law between the two countries, though. First, in the Dutch law there was the option of a “conditional” juridical measure (CTO) that functions both as a safety net to avoid a crisis and also as a juridical legitimation for professionals to intervene in situations in which a client was not motivated for care. Second, the need for treatment criterion in the Italian law around forced care restricts the situations in which juridical measures can be applied and enforces the idea (going back to Basaglia) that mental health care is responsible for care and not for custody.

#### The Esthetics of the Dance: Ideals Regarding Good Care

The choreographies in Trieste and Utrecht not only describe different care-control practices, they also reflect ideals about what is seen as good care around a crisis. In Trieste, the strong emphasis on people as part of a social network and creating continuity of care by providing care from a single team are key elements in what we could call a *relational* care-control choreography. Working on these relationships enables the team to “hear the whispers” of service users and thus to prevent a crisis. This is strengthened by the principle of open doors, which leads to a specific practice of controlling crisis situations in which the relationships are often intensified by staying close to someone in more or less intrusive ways and in which responsibility is shared: the more the service user is capable to handle and run his behavior, the less the service applies side-by-side forms of care-control. Care-control, then, is not a juxtaposition but a continuum—and moving along this continuum by engaging in relationships with the network is a way to avoid forced care. Going out for an ice cream, for instance, is not a form of coercion; however, in this way of caring the situation is indeed controlled.

In Utrecht, mental health care is both more specialized and more fragmented at the same time, with people referred to different teams depending on the specific situation. This means that in a situation of crisis it is of importance to connect these different expertise's. We therefore call this a care-control choreography *of connecting expertise*. In this choreography the ideal of respecting the individual autonomy of patients is central. This care vision gives clear directions on how to perform good care when a patient is motivated (making decisions based on informed consent and the agency of the patient) but does not give such a clear answer to the question about what to do when patients are not motivated for care or not willing to engage in contact. This was for instance, reflected in the discussions with the psychiatrist about when negotiation becomes manipulation.

As Pols points out (36), a strict division between care and control gives care givers little options to act between the two polarities of “doing nothing” from the idea of respecting individual autonomy and “applying coercion.” The division between care based on principles of individual autonomy or applying control by forced care than is not so much a continuum as a more or less strict line one has to cross, although we observed that this distinction between juridical and relational forms of control is not always clear-cut in practice.

## Discussion

Our analysis of the two care-control choreographies showed that a crisis is not only about the acute moment. Like in a dance choreography, there is an aspect of time and space: a crisis evolves in a specific situation following a certain time path. The time aspect directs attention to what happens before and after an acute moment and offers an alternative to a predominantly focus on risk (5). Broadening the perspective of crisis care to this wider timeframe is important as to enable care workers to “hear the whispers” that could signal the onset of a crisis and by able to prevent an escalation (37).

On both sides, ideally there are no forms of forced care. But in practice people do not always agree with interventions offered by professionals to avoid a crisis, or are not willing to engage in care at all. What to do? This question is addressed in different ways, for instance by developing guidelines for assertive outreach (38), and developing care models like Assertive Community Treatment (ACT) (39) and Fact (24). In this paper we addressed the question from an empirical ethical perspective: we described the daily practice of care and the values that are enacted in these. As Brodwin and Velprey (40) point out; ideas about control and constraint are connected to the “local shape of practice: the particular techniques, rationales, and limits of treatment that differ from site to site and one historical period to the next” (40), p. 525. In describing two of those specific practices in detail, we showed how care and control in practice go together in different ways. This relates to earlier work that points out how coercion and autonomy in practice are often interrelated (41, 42).

By contrasting the two field sites in Trieste and Utrecht as care-control choreographies we showed that what is perceived as good care around a crisis differ: In Trieste's *relational choreography* care is positioned as the opposite of exclusion and isolation. Professionals can intervene and persuade from within established relationships but the relationship should be maintained at all times: here, open doors are a prerequisite for good care. While forms of persuasion or interference are not problematized, strict forms of coercion such as a forced hospitalization are to be avoided as much as possible. There is a sense of unease when a relational approach fails and a forms of forced care are unavoidable.

In Utrecht's *choreography of connecting expertise*, the goods and the bads are distributed differently. The good involves respecting individual autonomy, supporting agency and making decisions based on the principles of informed consent. The bads to be avoided are interfering and taking over without a juridical ground. If care on the basis of informed consent does not work, then there is a “flip over” to juridical measures such as a CTO or forced hospitalization to control a crisis. This approach thus draws a more strict line between care and coercion and limits the options in between. As a result, in this choreography the legitimacy of cajoling, interfering or taking over is less clearly defined. But since care is relational (13, 43), caring *without* interfering is impossible. As a consequence, the relational way of working is also an important part of the daily practice of caring for a crisis in Utrecht, but can cause a sense of unease.

### Limitations

The findings of the study should be viewed in light of some limitations. First, the design of the research was limited to two teams to make in depth ethnographic fieldwork possible, but obviously this has consequences for the generalizability of the findings. The results describe how care-control around a crisis can be shaped in radically different ways and how both normativites (f.i. the concept of autonomy or relationality), organization of care and the way a crisis is identified are important factors in this. But these findings do not lead to “facts” that are applicable to community mental health in general. The findings are context bound descriptions, that we contrasted to learn about different ways of care-control around a crisis. What this can do is help to open up new ways of understanding care-and control and to formulate new questions in other settings. Future studies could bring to the fore other important aspects to improve the understanding of the relation between care and control and this could be helpful to determine indicators for good practices in situations around a crisis.

Second, as Malterud points out, (18) in qualitative (and maybe also in quantitative) research, the researchers position and perspectives has an effect on the research in different ways; on the questions asked, the methods chosen to collect data and the way they are interpreted. This positioning was addressed by being reflective on the role of the researcher, her connection to the field, the methodologies chosen en the theoretical framework that we used.

## Conclusion

As our fieldwork showed, care always means influencing and sometimes controlling the other, in more or less intense ways. In the discussion about care and coercion what is at stake is not how forms of control can be avoided at all times, but which forms of care-control are preferred in situations that are defined as (the onset of) a crisis. In the two choreographies we sketched, the connection between care and control is either described in terms of relationships or in terms of autonomy. This provides two choreographies of organizing care and handling a crisis in different ways. Contrasting these different ways of thinking about care-control, can help to open up more relational ways of thinking about caring for a crisis. Applying a strict boundary between care and control hinders the use of the relationship between caregiver and patient in care.

## Data Availability Statement

The datasets presented in this article are not readily available due to their containing information that could compromise the privacy of research participants. Requests to access the datasets should be directed to Christien Muusse, cmuusse@trimbos.nl.

## Ethics Statement

The studies involving human participants were reviewed and approved by the METC from VU University (FWA00017598), which has declared that the Medical Research Involving Human Subjects Act (WMO) does not apply to the study. Ethical permission was provided by the ethical commission of the TrimbosInstitute (TET). The patients/participants provided their written informed consent to participate in this study.

## Author Contributions

CM was responsible for data collection, performed the observations and interviews, analysis, and wrote the first draft of the paper. CM, HK, and JP were involved in the several rounds of analyses and provided comments on drafts of the paper. All authors contributed to the study design, concept development, and read and approved the final manuscript.

## Funding

This work was supported by Lister Sheltered Housing Utrecht; AMC Aspasia Travel Grant (JP).

## Conflict of Interest

The authors declare that the research was conducted in the absence of any commercial or financial relationships that could be construed as a potential conflict of interest.

## Publisher's Note

All claims expressed in this article are solely those of the authors and do not necessarily represent those of their affiliated organizations, or those of the publisher, the editors and the reviewers. Any product that may be evaluated in this article, or claim that may be made by its manufacturer, is not guaranteed or endorsed by the publisher.
